# Chronic urticaria and thyroid autoimmunity: a meta-analysis of case–control studies

**DOI:** 10.1007/s40618-022-01761-2

**Published:** 2022-02-18

**Authors:** D. Tienforti, F. Di Giulio, L. Spagnolo, C. Castellini, M. Totaro, M. Muselli, S. Francavilla, M. G. Baroni, A. Barbonetti

**Affiliations:** 1 Andrology Unit, Department of Life, Health and Environmental Sciences, University of L’Aquila, L’Aquila, Italy; 2 Epidemiology Division, Department of Life, Health and Environmental Sciences, University of L’Aquila, L’Aquila, Italy; 3 Neuroendocrinology and Metabolic Diseases, IRCCS Neuromed, Pozzilli, Isernia Italy

**Keywords:** Systematic review, Autoimmune thyroiditis, Anti-thyroperoxidase antibodies, Urticaria

## Abstract

**Purpose:**

Autoimmunity has been implicated in some patients with idiopathic chronic urticaria (CU). Because of the frequency of autoimmune thyroid diseases, their association with CU deserves special attention. We tested both the existence and the extent of an association between thyroid autoimmunity and CU.

**Methods:**

A thorough search of PubMed, Scopus, Web of Science, and Cochrane databases was performed. Studies reporting the positivity rate for anti-thyroperoxidase antibodies (TPOAbs) in people with (cases) and without CU (controls) were included. Quality of the studies was assessed by the Newcastle–Ottawa Scale. Between-study heterogeneity was assessed by Cochrane Q and *I*^2^ tests, and the odds ratio (OR) for TPOAbs positivity was combined using random-effects models.

**Results:**

Nineteen studies provided information about TPOAbs positivity on 14,351 patients with CU and 12,404 controls. The pooled estimate indicated a more than fivefold increased risk of exhibiting TPOAbs positivity in the group with CU (pooled OR 5.18, 95% CI 3.27, 8.22; *P* < 0.00001). Correction for publication bias had a negligible effect on the overall estimate (pooled adjusted OR: 4.42, 95% CI 2.84, 6.87, *P* < 0.0001). Between‑study heterogeneity was established (*I*^2^ = 62%, *P*_for heterogeneity_ = 0.0002) and when, according to meta‑regression models, a sensitivity analysis was restricted to the 16 studies with the highest quality scores, the OR for TPOAbs positivity rose to 6.72 (95% CI 4.56, 9.89; *P* < 0.00001) with no significant heterogeneity (*I*^2^ = 31%, *P*_for heterogeneity_ = 0.11).

**Conclusions:**

Patients with CU have a five-to-nearly sevenfold higher risk of displaying TPOAbs positivity. All patients with CU may well be offered a screening for thyroid autoimmunity.

**Supplementary Information:**

The online version contains supplementary material available at 10.1007/s40618-022-01761-2.

## Introduction

The term “urticaria” is widely used to define a skin manifestation characterized by the onset of itchy, fleeting wheals of variable size, shape, and distribution. These features arise from a series of pathophysiological events, including vasodilation, increased blood flow, and vascular permeability related to the activation and degranulation of mast cells, a process that can reflect both immunological and non-immunological mechanisms. Using a temporal criterion, urticaria can be classified into acute and chronic: in chronic urticaria (CU), manifestations occur daily or nearly daily and last for more than 6 weeks [1, 2]. In about 75% of patients, causes remain undefined, configuring the idiopathic CU [3]; however, many different pathogenic mechanisms have been proposed, including the occurrence of autoimmunity. Indeed, in some patients, CU may be associated with autoimmune diseases (AID) or, more generally, with positivity for autoantibodies [4, 5]. Among the possible associations, that between CU and thyroid autoimmunity has attracted interest, especially because of the epidemiological dimension of the autoimmune thyroid diseases.

The association between CU and Hashimoto’s thyroiditis was first described in 1983 by Leznoff and colleagues [6], who demonstrated the presence of anti-microsomal antibodies in 12% of patients suffering from idiopathic CU (i.e., twice the frequency of controls). Since then, many case–control studies have investigated such an association [6–11]; nevertheless, with very few exceptions [6, 12, 13], these reports have the major limitation of a low sample size and produced inconclusive results. While most studies found a significantly higher risk of thyroid autoimmunity in CU [6, 8, 12–19], some authors have found no association [20–27], while others have even documented a negative association between the two conditions [28]. Intriguingly, autoimmune thyroid diseases can also be associated with other skin disorders with autoimmune pathogenesis, namely non segmental vitiligo, where anti-thyroperoxidase antibodies (TPOAbs) can be detected [29].

On this basis, we carried out a systematic review and meta-analysis of the available case–control studies to comprehensively assess the overall risk of thyroid autoimmunity in people with diagnosis of CU.

## Methods

The meta-analysis was conducted according to the Cochrane Collaboration and the Preferred Reporting Items for Systematic Reviews and Meta-Analyses (PRISMA) statement [30]; it also complies with the guidelines for Meta-Analyses and Systematic Reviews of Observational Studies (MOOSE) [31]. PRISMA and MOOSE Checklists have been presented as Supplementary Table 1 and Supplementary Table 2, respectively.

The study protocol was registered in the international prospective registry for systematic reviews (PROSPERO) with registration number: CRD42021274422.

### Systematic search strategy

A systematic search was performed in PubMed, SCOPUS, Web of Science, and Cochrane Library, including the following free and vocabulary terms: “thyroid autoimmunit*”, “autoimmune thyroid disease*”, “TPOAb*”, “anti-thyroperoxidase antibod*”, “urticaria”, using the Boolean functions AND/OR. The search was restricted to English language, published up to December 2021. If it was not clear from the abstract whether the study contained relevant data, the full text was retrieved. The reference lists of the identified papers were also scrutinized to find possible additional pertinent studies.

### Inclusion/exclusion criteria

The outcome of interest was the relationship between CU and positivity for TPOAbs. The eligibility criteria were the following: (1) observational case–control studies, enrolling patients with (cases) and without CU (controls); (2) availability of odds ratio (OR) for having TPOAbs positivity or data for its calculation in both groups. Duplicates were rigorously checked and removed. Studies with missing/incomplete or unsuitable data or lacking to assess the outcome of interest were excluded. Three independent reviewers (D.T., F.D.G., and L.S.) assessed the eligibility of each selected paper; any disagreement was resolved via discussion involving a fourth reviewer (A.B.).

### Data extraction

Data were extracted from the selected articles by including the first author, publication year, geographic region, mean age of participants, the number of events (TPOAbs positivity), and the total number of participants in cases and controls. Additional information, such as TPOAbs assay methods and positivity cut-offs, male-to-female ratio, levels of thyroid stimulating hormone (TSH), percentages of patients with thyroid dysfunction (hypo- and hyperthyroidism), and/or severe urticaria (angioedema), was also extracted when available. Where data were missing or inconsistent, the authors were contacted to obtain the necessary information.

### Assessment of study reporting quality

The quality of the studies was assessed using the Newcastle–Ottawa Scale (NOS) [32]. The score was calculated from 0 to 9, and studies with a score of at least 7 were considered to be of good quality. The evaluation was performed independently by three reviewers (D.T., C.C., and M.T.), involving a fourth reviewer (A.B.) to solve any discrepancies in judgment.

### Statistical analysis

The relationship between thyroid autoimmunity and CU was assessed by calculating the aggregate OR (95% CI) for TPOAbs positivity comparing cases and controls. Mantel–Haenszel estimates were combined in a random-effects model [33], which is more conservative than the fixed-effects model [34], because it accounts for heterogeneity in the calculation of the overall estimate. Study-specific estimates, their precision, and the presence of heterogeneity among them were visualized using forest plots. The Cochran’s *χ*^2^ (Cochran’s Q) and *I*^2^ tests were carried out to analyze statistical heterogeneity between the results of different studies. *I*^2^ > 50% and/or *P* < 0.05 indicated substantial heterogeneity.

Publication bias was explored through the funnel plot [35] and the Egger’s test [36]. To correct for publication bias, the Duval and Tweedie’s ‘trim-and-fill’ analysis was carried out [37], as previously reported [38]. Briefly, in the presence of asymmetric funnel shape, this test detects putative missing studies to rebalance the distribution and provides an adjusted pooled estimate taking the additional studies into account, thus correcting the analysis for publication bias.

To detect possible sources of the between-study heterogeneity, available covariates that could affect the estimates were included in linear meta-regression models.

Data were analyzed using the R statistical software (version 3.6.3; R Foundation for Statistical Computing, Vienna, Austria), using the “metafor” package, and the Review Manager (RevMan) of the Cochrane Library (version 5.3; The Nordic Cochrane Centre, The Cochrane Collaboration, Copenhagen, Denmark).

## Results

### Selection of studies

**Fig. 1:**
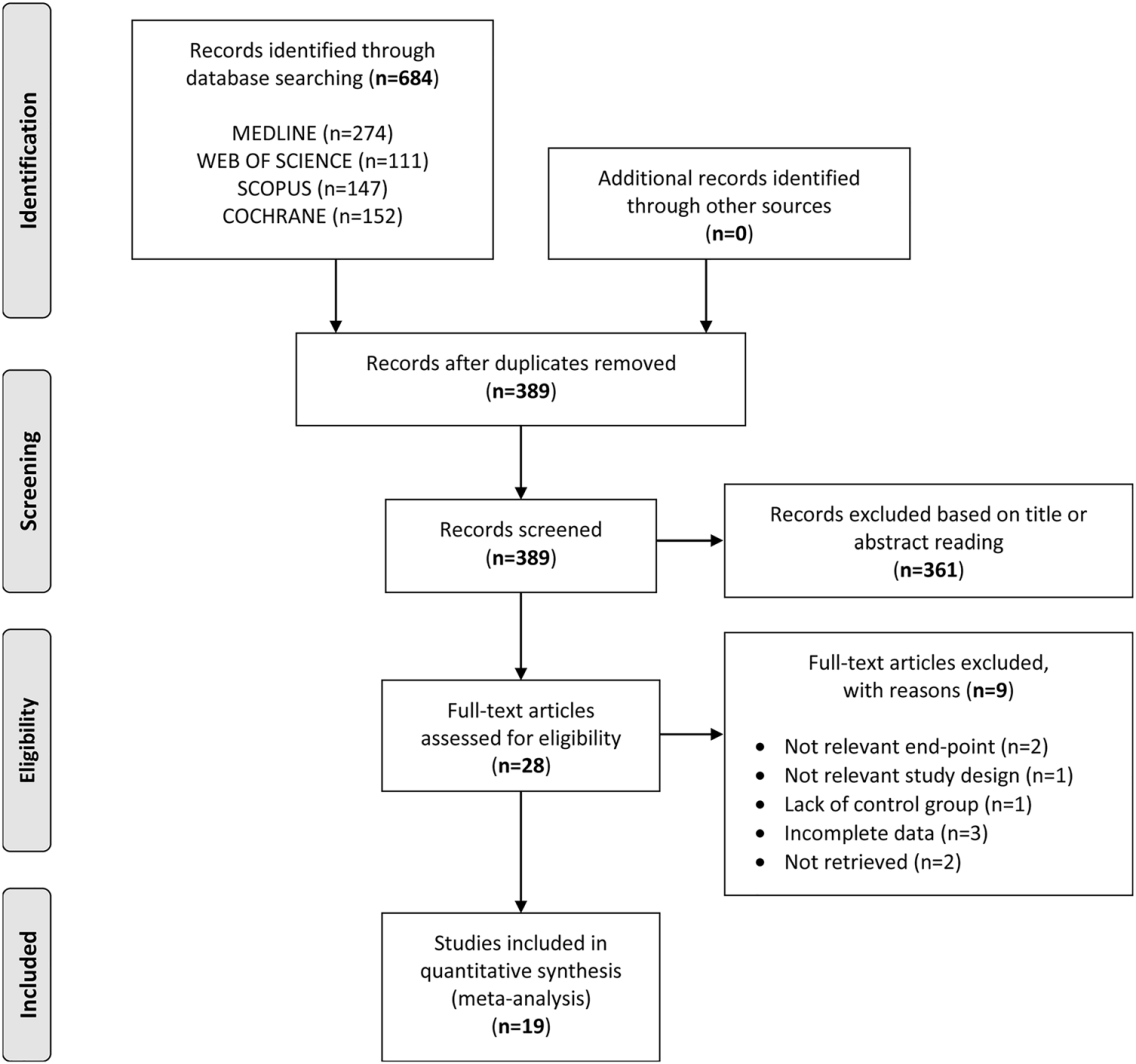
Flow diagram showing an overview of the study selection process

The electronic search yielded a total of 684 articles. After removal of duplicates, 389 articles were obtained, of which 361 were excluded, because they were deemed irrelevant based on title and/or abstract reading. Thus, as shown in Fig. 1, a total of 28 articles were identified, of which 19 met the inclusion criteria [6, 8, 12–28].

Details of the main characteristics of the papers included in the quantitative analysis are shown in Table 1.

**Table 1:** Details of the main characteristics of the 19 articles included in the quantitative analysis

Study	Region	Mean age (years)	CU group: TPOAbs + /total	Control group: TPOAbs + /total	TPOAbs assay methods	TPOAbs positivity cut-off	Females (%)	Mean TSH levels (mU/L)	HypoT (%)	HyperT (%)	AE (%)
Leznoff, 1983 [6]	Canada	NR	17/140	27/477	HA	≥ 1:100	70.0	NR	2.1	2.1	NR
Turktas, 1997 [24]	Turkey	37.0	9/94	4/80	IRMA	≥ 100 IU/ml	72.0	NR	3.2	1	70.2
Ryhal, 2001 [8]	USA	48.0	5/25	0/75	NR	NR	NR	NR	NR	NR	NR
Verneuil, 2004 [25]	France	43.0	8/45	1/30	ELISA	> 25 IU/ml	78.0	NR	0	0	33.3
Cebeci, 2006 [15]	Turkey	40.0	23/140	8/181	NR	NR	73.0	1.9	0.7	1.4	42.9
Aamir, 2008 [14]	Pakistan	37.0	13/30	0/30	HA	≥ 1:100	100.0	1.8	10	NR	NR
Nuzzo, 2011 [19]	Italy	31.5	12/54	5/108	NR	> 110 IU/ml	NR	NR	18.5	NR	18.5
Al-Balbeesi, 2011 [20]	Saudi Arabia	41.0	18/68	1/22	HA	≥ 1:100	90.0	NR	NR	NR	NR
Confino-Cohen, 2012 [12]	Israel	45.0	598/12778	54/10714	NR	NR	66.0	NR	10	2.6	NR
Wan, 2013 [26]	Taiwan	NR	5/60	0/40	NR	NR	NR	0.6	0	0	NR
Yadav, 2013 [27]	India	33.0	14/80	2/40	ELISA	NR	56.0	NR	NR	NR	NR
Alpay, 2013 [21]	Turkey	46.0	6/50	2/50	NR	NR	78.0	NR	6	NR	NR
Ghaffari, 2013 [22]	Iran	29.0	5/78	2/67	NR	NR	70.5	NR	1.3	NR	30.8
Cho, 2013 [28]	USA	39.0	3/27	4/19	ECLIA	≥ 35 IU/ml	85.0	1.1	NR	NR	NR
Okba, 2015 [23]	Egypt	36.0	20/80	4/40	IFAT	NR	77.5	4.6	18.8	13.7	NR
Diaz Angulo, 2015 [13]	Spain	44.0	70/343	5/282	EIA	> 90 IU/ml	70.5	NR	3.8	1.2	NR
Magen, 2016 [18]	Israel	34.0	13/41	3/44	NR	> 75 IU/ml	68.0	2.3	NR	NR	NR
Kasumagic-Halilovic, 2017 [17]	Bosnia	40.0	12/70	1/70	ECLIA	> 34 IU/ml	57.0	NR	NR	NR	NR
Czarnecka-Operacz, 2017 [16]	Poland	42.0	32/148	0/35	RIA	> 34 IU/ml	98.0	1.7	2.7	NR	54

*TPOAbs* anti-thyroid peroxidase antibodies, *AE* angioedema, *CU* chronic urticaria *ECLIA* electrochemiluminescence immunoassay, *EIA* enzyme immunoassay, *ELISA* enzyme linked immunosorbent assay, *HA* hemoagglutination, *HyperT* Hyperthyroidism, *HypoT* Hypothyroidism, *IFAT* indirect immunofluorescence antibody test, *IRMA* Immunoradiometric assay, *IU* international units, *NR* not reported, *RIA* radioimmunoassay, *TSH* thyroid stimulating hormone

### Quality of included studies

The quality rating of the studies, based on the NOS, is presented in Table 2. The score ranged from 5 to 9. Sixteen studies were judged to be of good quality, having been assigned a score of ≥ 7, whereas 3 articles were of moderate quality. In particular, in the studies by Leznoff and colleagues [6] and Cho et al. [28], a possible bias arose from the definition of controls, as their clinical characteristics were not clearly defined.

**Table 2:** Newcastle–Ottawa Scale for the quality assessment of case–control studies

Study	Selection				Comparability		Exposure			Total
	Definition of exposure	Representativeness	Selection of controls	Definition of controls	On age	On other risk factors	Assessment of exposure	Same methods of ascertainment for cases and controls	Nonresponse rate	
Aamir, 2008 [14]	1	1	1	1	1	0	1	1	1	8
Al-Balbeesi, 2011 [20]	1	1	1	1	1	0	1	1	1	8
Alpay, 2013 [21]	1	1	0	0	1	0	1	1	1	6
Cebeci, 2006 [15]	1	1	1	1	1	1	1	1	1	9
Cho, 2013 [28]	1	1	0	0	0	0	1	1	1	5
Confino-Cohen, 2012 [12]	1	1	1	1	1	1	1	1	1	9
Czarnecka-Operacz, 2017 [16]	1	1	1	1	1	1	1	1	1	9
Diaz Angulo, 2015 [13]	1	1	1	1	1	1	1	1	1	9
Ghaffari, 2013 [22]	1	1	1	1	1	1	1	1	1	9
Kasumagic-Halilovic, 2017 [17]	1	1	1	1	1	1	1	1	1	9
Leznoff, 1983 [6]	1	1	1	0	0	0	1	1	0	5
Magen, 2016 [18]	1	1	0	1	1	1	1	1	1	8
Nuzzo, 2011 [19]	1	1	1	1	1	1	1	1	1	9
Okba, 2015 [23]	1	1	0	1	1	1	1	1	1	8
Ryhal, 2001 [8]	1	1	1	1	0	0	1	1	1	7
Turktas, 1997 [24]	1	1	0	1	1	1	1	1	1	8
Verneuil, 2004 [25]	1	1	1	1	1	1	1	1	1	9
Wan, 2013 [26]	1	1	0	1	1	1	1	1	1	8
Yadav, 2013 [27]	1	1	1	1	1	1	1	1	1	9

### Summary of results

**Fig. 2:**
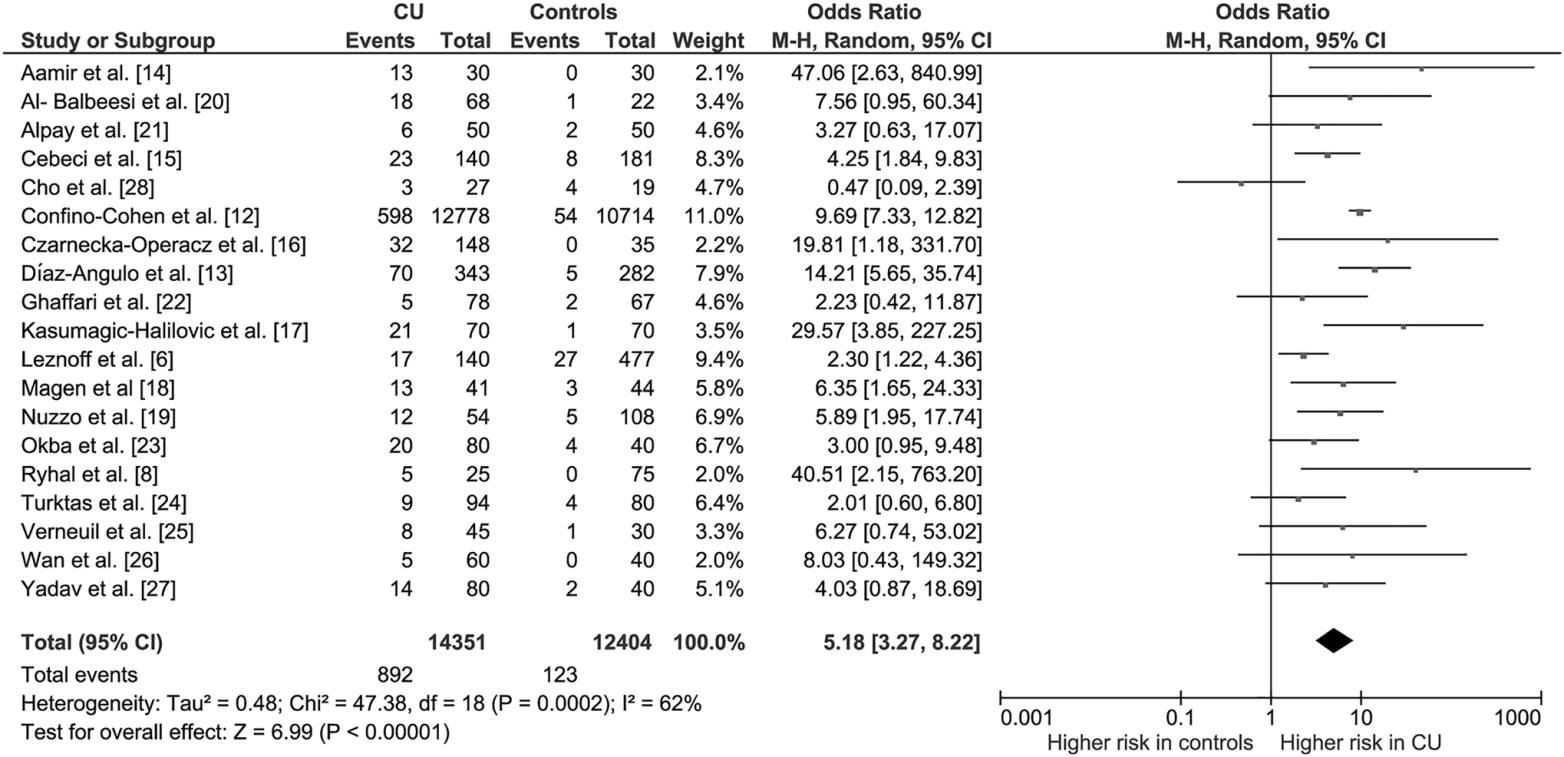
Forest plot of odds ratios for thyroid autoimmunity in subjects with and without chronic urticaria. Diamond indicates the overall estimate (width of the diamond represents 95% CI). Boxes indicate the weight of individual studies in the pooled result. *CI* confidence interval, *CU* chronic urticaria, *df* degrees of freedom, *M–H* Mantel–Haenszel

Overall, the 19 studies included in the meta-analysis provided information on 14,351 patients with CU (cases) and 12,404 subjects without CU (controls), resulting in an overall crude rate of TPOAbs positivity of 3.8%. The pooled OR indicated a more than fivefold increased risk of exhibiting TPOAbs positivity in the group with CU (pooled OR 5.18, 95% CI 3.27, 8.22; *P* < 0.00001; Fig. 2). The analysis revealed a significant between-study heterogeneity (*I*^2^ = 62%, *P*_for heterogeneity_ = 0.0002).

### Publication bias

**Fig. 3:**
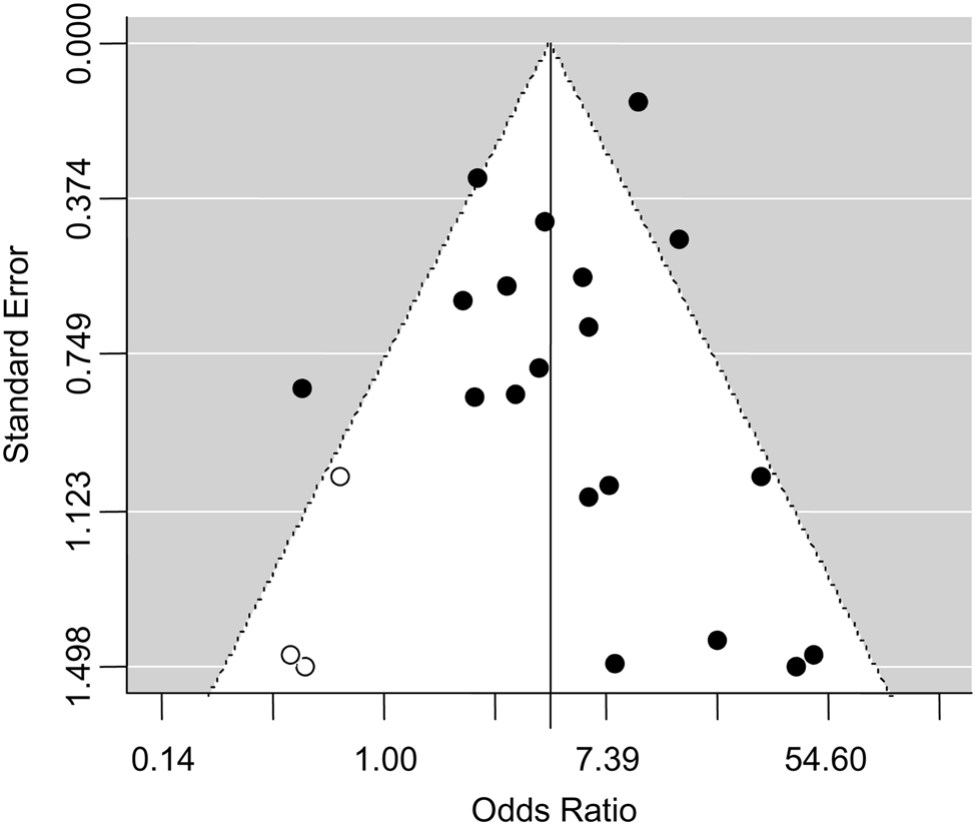
Funnel plot of case–control studies of the association between chronic urticaria and thyroid autoimmunity. Trim-and-fill test identified three putative missing studies (white circles) on the left side of the distribution

The asymmetrical shape of the funnel plot (Fig. 3) suggested the presence of a publication bias. Although the Egger’s test revealed a not significant degree of asymmetry (*t* = − 1.05, *P* = 0.31), the trim-and-fill test identified three putative missing studies on the left side of the distribution (Fig. 3). However, the inclusion of these additional studies resulted in a negligible effect on the overall estimate (pooled adjusted OR: 4.42, 95% CI 2.84, 6.87, *P* < 0.0001; *I*^2^ = 58.7%, *P*_for heterogeneity_ < 0.0001).

### Heterogeneity analysis

As between-study heterogeneity in the pooled analysis was found (Fig. 2), linear meta-regression analyses were carried out to detect possible sources of the variability. Among available covariates that could affect the estimates, including publication year, overall prevalence of thyroid autoimmunity, male-to-female ratio, TSH levels, rate of thyroid dysfunction (hypo- and hyperthyroidism), rate of severe urticaria (angioedema), and NOS quality score, only this latter contributed significantly to the heterogeneity (Supplementary Fig. 1): a higher study NOS score was significantly associated with the report of a higher risk for thyroid autoimmunity in patients with CU compared to controls (β-coefficient = 0.36, 95% CI 0.15, 0.58; *P* = 0.001).

**Fig. 4:**
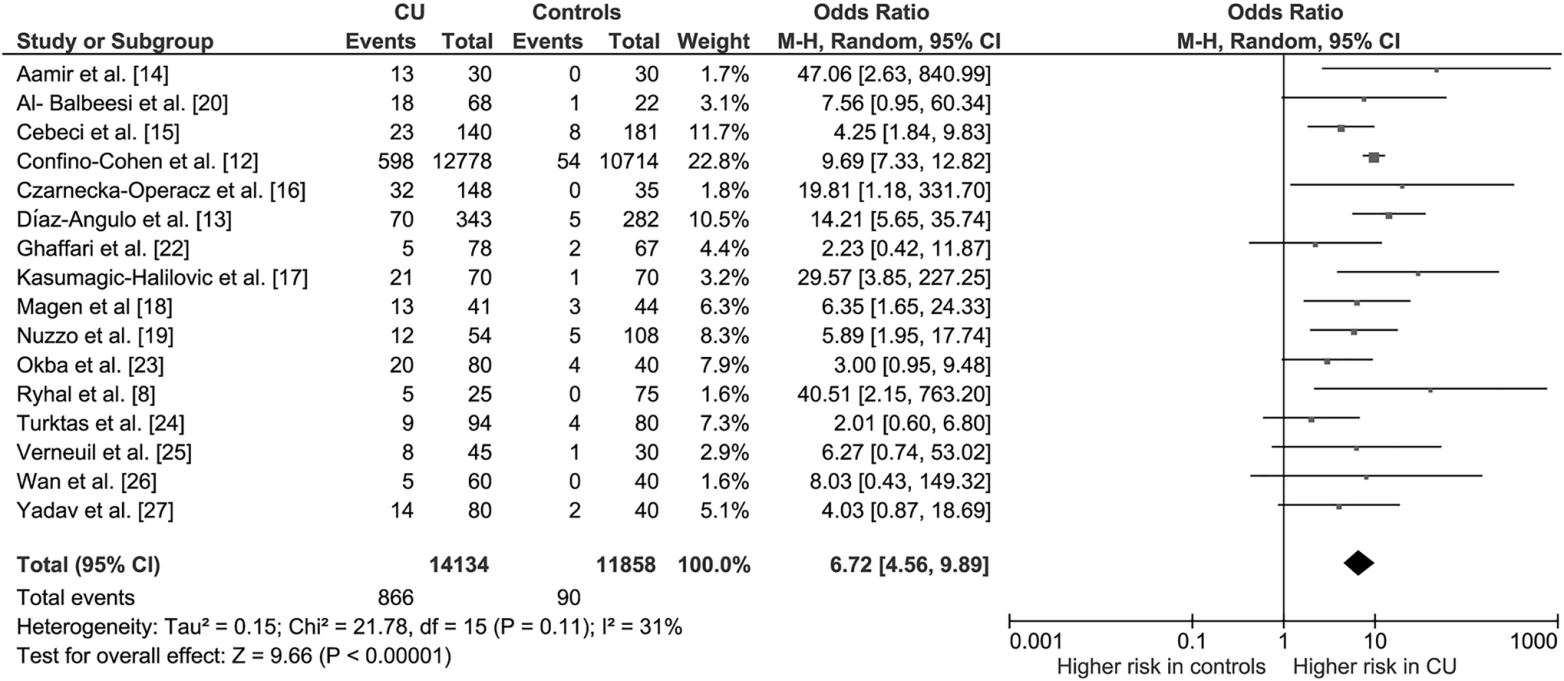
Sensitivity analysis based on the quality score at the Newcastle–Ottawa Scale (NOS): only studies with NOS score ≥ 7 were included. Forest plot of odds ratios for thyroid autoimmunity in subjects with and without CU. Diamond indicates the overall estimate (diamond width represents 95% CI). Boxes indicate the weight of individual studies in the pooled result. *CI* confidence interval, *CU* chronic urticaria, *df* degrees of freedom, *M–H* Mantel–Haenszel

As shown in Fig. 4, when, according to the meta-regression results, a sensitivity analysis was restricted to studies with an NOS score ≥ 7, the OR for TPOAbs positivity rose to 6.72 (95% CI 4.56, 9.89; *P* < 0.00001) with decrease of heterogeneity to a no longer significant degree (*I*^2^ = 31%, *P*_for heterogeneity_ = 0.11).

## Discussion

The genesis of spontaneous CU has been considered idiopathic for many years, but the identification of autoantibodies in some patients has given rise to the new concept of “autoimmune urticaria” [39]. In this view, as immune dysregulation factors are shared by both urticaria and autoimmune thyroiditis [40, 41], it might be expected that the two entities may often coexist in the same patient. This association, albeit widely investigated, still remains somehow uncertain due to a number of limitations of the studies, including the small sample sizes, heterogeneity among the results, and the different diagnostic criteria used to define thyroid autoimmunity.

In the present meta-analysis of 19 rigorously selected case–control studies, the diagnosis of CU was associated with an approximately fivefold higher risk of exhibiting positivity for TPOAbs, a marker of chronic autoimmune thyroiditis. In the analysis restricted to studies with higher quality scores, the OR of the association increased to 6.72 (95% CI 4.56, 9.89) with a not significant heterogeneity.

The mechanisms underlying the association between CU and thyroid autoimmunity are not fully elucidated. An immune-regulatory role of TSH would be supported by some evidence on the effect of levothyroxine treatment that, in some studies, led to improvements of urticaria clinical features [7, 42, 43], an effect believed to be mediated by the decrease in TSH levels [42]. However, other studies failed to demonstrate the efficacy of levothyroxine [44, 45], and even when an effect has been found, it was challenged by the limited sample size of the study populations together with the lack of a control group. Although the studies included in this meta-analysis largely lacked information about levothyroxine treatment, the role of TSH seems to be unlikely as meta-regression analyses showed no significant influence of either TSH levels or the rate of thyroid dysfunction (hypo- or hyperthyroidism) on the association under investigation.

One of the pieces of evidence that mostly supports the autoimmune genesis of CU is based on the observation that 45% and 55% of patients with CU display anti-IgE and anti-FcεRI antibodies, respectively, whose titre exhibits a well-documented correlation with the positivity of the autologous serum skin test [46]. The evidence of an autoimmune pathogenesis of CU creates the basis for theorizing the existence of common immunopathologic mechanisms shared by CU and autoimmune thyroiditis [40, 41]. One mediator called into play is the interleukin 6 (IL6). In vitro experiments have shown that IL6 increases endothelial permeability, a key mechanism in the pathogenesis of urticaria [47]. The levels of this cytokine are indeed higher in patients with CU than in healthy controls and decrease with remission of urticaria [48]. Interestingly, high circulating levels of IL6 have been also reported in Hashimoto’s thyroiditis where they positively correlate with the number of Th22 lymphocytes, which in turn is related to the TPOAbs titre [49, 50]. Another possible immunopathologic link underlying the association between autoimmune thyroiditis and CU could lie in a defective activity of regulatory T cells (Tregs). Tregs are T lymphocytes with suppressor properties on effector immune cells, and a decrease in number and/or functionality of Tregs was associate with a variety of autoimmune disorders [51]. Interestingly, decreased percentage and activity of Tregs have been demonstrated both in CU [52–54] and autoimmune thyroid diseases [55, 56]. Unfortunately, the lack of information on these immuno-molecular factors hindered quantitative analyses to clarify their possible pathogenic role.

This meta-analysis has some limitations. First, some potentially relevant studies were not included because of incomplete or unsuitable data. In this regard, only articles published in English language retrievable from well-recognized electronic databases were screened for eligibility. Nevertheless, when the analysis was restricted to TPOAbs, we included more studies than in the only other previously published meta-analysis on the topic [57]. Second, different criteria for the diagnosis of thyroid dysfunction could have made it difficult to compare the studies when the rate of hypothyroidism and hyperthyroidism was analyzed in meta-regression. Moreover, although TPOAbs represent a sensitive marker of autoimmune thyroid disease, their positivity is not a sufficient criterion for the diagnosis of chronic autoimmune thyroiditis and there may be also cases of autoimmune thyroid disease with negative TPOAbs [58]. On the other hand, TPOAbs positivity can be found also in subacute thyroiditis, as well as in non-thyroid autoimmune disorders. Unfortunately, available studies were largely lacking in information about thyroid ultrasonographic features that could have helped in the correct diagnostic framing. As another major limitation, many of the included studies may not be fully comparable due to the different assay methods and cut-offs used to identify TPOAbs positivity (Table 1). These differences may have affected the accuracy of the overall estimate. Finally, unadjusted data were extracted and pooled in our quantitative synthesis: availability of point estimates corrected for confounders would have made it possible to produce a more representative pooled estimate of the true association between CU and thyroid autoimmunity. To partially overcome this limitation, meta-regressions were conducted that did not demonstrate a significant role as a source of heterogeneity of several possible confounding factors, except for the methodological quality, highlighting that the studies with lower risk of bias found the strongest associations.

In conclusion, people with CU have a five-to-nearly sevenfold higher odd of exhibiting TPOAbs positivity. From a clinical point of view, independently of the pathogenesis underlying such an association, this finding points to the opportunity to perform a screening for thyroid autoimmunity in the presence of CU. This approach could allow identification of patients with chronic autoimmune thyroiditis still in euthyroidism who could benefit from monitoring of TSH levels over time.

## Supplementary Information

Below is the link to the electronic supplementary material.

Supplementary file1 (DOC 86 KB)

Supplementary file2 (DOC 72 KB)

Supplementary file3 (TIF 10368 KB) Supplementary Figure 1. Meta-regression bubble plot: odds ratio for having thyroid autoimmunity as a function of the quality score of the studies at the Newcastle-Ottawa Scale (NOS). The predicted effects (solid line) with corresponding confidence intervals (dashed lines) are also shown. Odds ratio values below 1 indicate a lower risk of thyroid autoimmunity in people with chronic urticaria; Odds ratio values above 1 indicate a higher risk of thyroid autoimmunity in people with chronic urticaria.
