# Supplementary figures and images for: Chronic urticaria and thyroid autoimmunity: a meta-analysis of case–control studies
Source: J Endocrinol Invest. 2022 Feb 18;45(7):1317–26. doi: 10.1007/s40618-022-01761-2 (PMC9184403; doi:10.1007/s40618-022-01761-2)

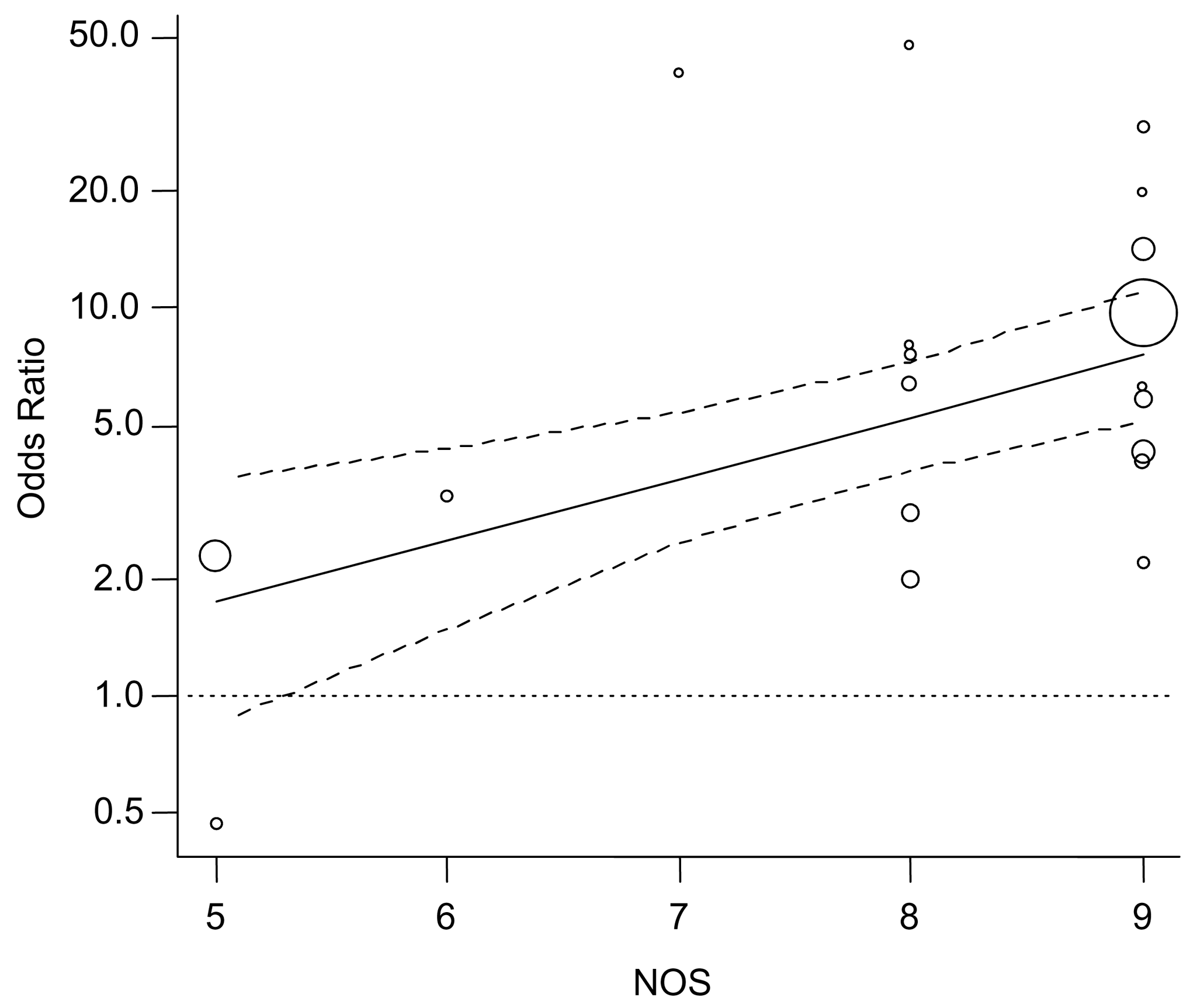

Supplement: Supplementary file 3 — Supplementary file3 (TIF 10368 KB) Supplementary Figure 1. Meta-regression bubble plot: odds ratio for having thyroid autoimmunity as a function of the quality score of the studies at the Newcastle-Ottawa Scale (NOS). The predicted effects (solid line) with corresponding confidence intervals (dashed lines) are also shown. Odds ratio values below 1 indicate a lower risk of thyroid autoimmunity in people with chronic urticaria; Odds ratio values above 1 indicate a higher risk of thyroid autoimmunity in people with chronic urticaria. [file 40618_2022_1761_MOESM3_ESM.tif]
